# Synthesis of Ion-Exchange Catalysts by Introduction of Fluorinated Ponytails into Novel Mesoporous Polymers

**DOI:** 10.3390/ma16103808

**Published:** 2023-05-18

**Authors:** Chiara Dalla Valle, Francesco Sandri, Marco Zecca, Federico Rastrelli, Sandro Campestrini, Paolo Centomo

**Affiliations:** Dipartimento di Scienze Chimiche, Università degli Studi di Padova, Via Marzolo 1, 35131 Padova, Italy

**Keywords:** polymer functionalization, fluoropolymers, heterogeneous catalysis, esterification

## Abstract

A novel synthetic procedure for the functionalisation of styrenic cross-linked polymers with perfluorinated acyl chains has been reported. The effective significant grafting of the fluorinated moieties is supported by {^1^H}-^13^C and {^19^F}-^13^C NMR characterisations. This kind of polymer appears promising as catalytic support for a variety of reactions requiring a highly lipophilic catalyst. Indeed, the improved lipophilicity of the materials resulted in enhanced catalytic properties of the corresponding sulfonic materials in the reaction of esterification of a solution in a vegetable oil of stearic acid with methanol.

## 1. Introduction

Styrenic resins are a well-established class of materials widely used both in academic and technological practice [1,2,3,4], especially for separations and catalysis. The functionalisation of aromatic compounds is extensively reported and makes styrenic resins versatile materials for catalytic purposes [2]. In addition to the introduction of active chemical groups, such as sulfonic groups for ion exchange or acid catalysis, they can be properly functionalised to tune their chemical and/or physical properties and the overall behaviour of the materials [2,5,6]. This can be very important in a number of applications of polymer matrices, such as their use of templates for the production of inorganic nanoparticles [7,8,9].

In a previous investigation on the esterification of fatty acids, it was observed that the introduction of lipophilic domains inside mesoporous silica materials bearing propylsulfonic acid sites enhances the reaction between reagents with very different lipophilicity (i.e., fatty acid and alcohol), by facilitating the diffusion of the lipophilic substrate towards the hydrophilic sulfonic groups [10]. A similar approach can be used for styrenic sulfonated resins, widely used as acid catalysts in a number of reactions involving substrates with a mismatch in their hydrophilic/hydrophobic character [2,11]. For instance, a gel-type cross-linked styrene-divinylbenzene copolymer (2% cross-linked) was successfully acylated (50–80% of aromatic rings) with acyl chlorides containing two (acetyl chloride), four (butyryl chloride), or eight (octanoyl chloride) carbon atoms linearand subsequently sulfonated [12,13]. The resulting acid catalysts showed promising behaviour in the esterification of an oil solution of stearic acid with methanol, an interesting model reaction in that fatty acid methyl esters (FAMEs) are the constituents of biodiesel [14,15]. When the amount of methanol in the reaction mixture was low (5%, *w*/*w*), sulfonic resins gave a conversion of about 5% due to the low swelling degree. On the contrary, acylated catalysts showed greater conversions due to the assisted absorption of the lipophilic reagent inside the polymer framework. On the basis of these results, the introduction of perfluoro-acylic chains could be potentially interesting thanks to their higher lipophilic character with respect to their non-fluorous analogs. Moreover, the solubility of gases, such as hydrogen and oxygen, in fluorinated solvents, which is remarkably higher than in organic solvents [16], could make this material attractive for applications entailing the diffusion of gases into a solid phase, such as supported metal catalysts. In this work, we report on the preparation of acidic ion-exchange catalysts (IECs) based on a gel-type sulfonated polystyrene-divinylbenzene resin and on mesoporous polydivinylbenzene, both bearing highly lipophilic perfluorinated butylryl groups. A careful solid-state NMR characterisation showed the effectiveness of the functionalisation, the effect of which was preliminarily tested in the esterification of stearic acid with methanol catalysed by the gel-type IEC.

## 2. Experimental

A gel-type styrene-co-divinylbenzene resin with a nominal divinylbenzene (DVB) content of 2% mol (provided by Spolchemie, Usti nad Labem, Czech Republic) was used as the polymer substrate. Reagents and solvents were purchased from Sigma-Aldrich (St. Louis, MO, USA) and used as received.

### 2.1. Synthesis of Mesoporous Polydivinylbenzene

Poly-divinylbenzene was synthesised according to ref. [17]. In a typical preparation of the pDVB material, 6.0 g of divinylbenzene (technical grade 80%), 60 mL of tetrahydrofuran (THF), 165 mg of 2,2′ azobis(2-methylpropionitrile) (AIBN), the initiator, and 6 mL of distilled water were introduced, under magnetic stirring, in a Teflon vessel with a capacity of 85 mL. The stirring was maintained for three hours. The vessel, containing the pale-yellow polymerisation mixture, was transferred in a closed autoclave and let to react at 110 °C for 48 h under autogenic pressure (2.1 bar). After cooling to room temperature, a white gelatinous monolith was recovered from the autoclave and left to dry at room temperature for ten days.

### 2.2. Acylation with Perfluorobutyryl Chloride

In a typical experiment, ca. 2 g of resin was let to fully swell in a round-bottomed flask with 20 mL of CHCl_3_. Ca. 4 g of aluminium chloride, ca. 8 g of perfluorobutyryl chloride (35 mmol), and 20 mL of CHCl_3_ were added to the suspension under mild magnetic stirring. Stirring was maintained at room temperature for 48 h, and 150 mL of HCl 1.2 M were added. After moderate stirring at room temperature for a further 24 h CHCl_3_ was removed with a rotary evaporator, and 100 mL of HCl 1.2 M were added to the polymer suspension. The product was recovered by filtration under reduced pressure on a sintered glass filter and washed with distilled water, a water/THF mixture (1/1, *v/v*), THF, CHCl_3_, and CH_2_Cl_2_ (300 mL each).

### 2.3. Sulfonation with Concentrated Sulfuric Acid

Ca. 2 g of the acylated polymer was let to swell overnight in a suitable volume of 1,2-dichloroethane in a jacketed glass reactor equipped with a reflux condenser. 50–100 mL of 98% H_2_SO_4_ (acid:solvent, 5:1 *v*/*v*) were slowly added to the swollen polymer. The mixture was stirred at 80 °C for 3 h. After cooling to room temperature, the reaction mixture was diluted by adding 30 mL of a 10 M solution of sulfuric acid, keeping the temperature low with cold water running in the jacket of the reactor. Dilution continued similarly by further consecutive additions of 30 mL of 5.0, 2.5, 1.0, and 0.1 M solutions, respectively, of sulfuric acid. Finally, the resin was recovered by filtration, washed with distilled water up to the neutral pH of the liquor and dried at 110 °C. The ion exchange capacity of the polymeric materials was determined by treatment with a suitable (excess) amount of 0.1 M NaOH and subsequent back-titration with 0.1 M HCl, according to ref. [13].

### 2.4. Esterification of Stearic Acid

A total of 72 g of sunflower oil and 8 g of stearic acid (SA) were transferred into a Schott-Duran glass bottle and magnetically stirred under mild heating, up to the dissolution of stearic acid. The mixture was let to cool down to room temperature and stand overnight. The day after, 500 mg of catalyst were swollen in ca. 5 mL of methanol for two hours. After the addition of the catalyst and of 20 g of anhydrous methanol to the mixture of SA and oil, the bottle was closed with a Teflon cap equipped with an Allihn condenser, a port for a thermocouple, and a glass line for sampling the reaction mixture with a syringe. The bottle, which was used as a glass reactor, was heated to 150 °C with an oil bath. The time when the reaction mixture reached 65 °C was taken as the starting point of the reaction. The reaction was stopped after 8 h, and samples of the reaction mixture (ca. 1 g) were periodically withdrawn with a syringe to monitor its progress.

### 2.5. Solid-State NMR

NMR spectra in the solid-state were collected on a Varian 400 equipped with a narrow bore, triple resonance T3 MAS probe spinning 4 mm rotors and operating at ^1^H, ^19^F and ^13^C frequencies of 400.36, 376.67, and 100.68 MHz, respectively. The nominal temperature of the probe was always set to 298 K. ^13^C CP-MAS spectra were acquired at 10 kHz MAS with 1200 scans, and a repetition delay of 3 s: more details are provided in the text below each spectrum. The chemical shifts were referenced against the CH_2_ resonance observed for adamantane at 38.48 ppm with respect to the signal for neat TMS. In order to homogenise the samples, resin beads were ground to obtain a fine powder prior to insertion into the rotor.

### 2.6. DFT Calculations

Calculations were run using the Gaussian 09 software [18]. Geometries of the selected oligomeric sub-systems have been optimised in the gas phase at the B3LYP level of theory. Nuclear shieldings were computed at the B3LYP/cc-pVTZ level of theory [19,20]. Chemical shifts were calculated as δ = σ_ref_ − σ, where σ_ref_ is the shielding constant of ^1^H or ^13^C in the reference compound TMS or ^19^F in reference compound CFCl_3_.

## 3. Results and Discussion

A gel-type cross-linked polymer (styrene-DVB copolymer, 2% mol DVB) was chosen as the first base resin for the introduction of perfuloroacylic chains. Gel-type resins are known to have no porosity in the dry state and a relatively regular microporosity in the swollen state [21]. For these reasons, their BET characterisation is of very little use, if any, on the one hand, but on the other, they are most suited to assess the effects of their functionalisation. As catalytic supports, they must swell to an extent high enough to allow diffusion inside the catalyst, and therefore, their performance is strongly affected by their swelling degree under reaction conditions [2,22]. This critically depends on the match between the properties of the reaction medium (e.g., its polarity), which must also swell the support and the support itself [9,23]. As the properties of the support can be changed by introducing different functional groups, they can be tailored to optimise the reaction conditions for the desired catalytic reaction. Gel-type resins are the most sensitive to this kind of effects, hence most suited to assess them. Similar effects are in action also during the functionalisation of the resins; hence perfluorobutyryl chloride was selected for the acylation of the resins as the best balance between the highest possible effect of “lipophilization” of the resin (the longer the acylic chain, the better) and the steric hindrance which is expected to impair the acylation reaction (the longer the acylic chain, the worse).

**Scheme 1:**
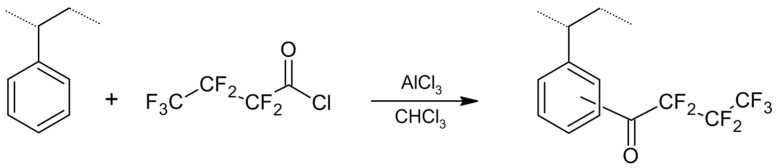
Acylation of a generic styrenic polymer with perfluorobutyryl chloride.

The procedure for the acylation of styrenic resins with hydroacyl chlorides in CS_2_ [13] proved ineffective because perfluorobutyryl chloride is insoluble in this solvent. The complete dissolution of the required amount of perfluorobutyryl chloride was achieved in chloroform, and a material hereafter referred to as Gel_C_4_F_7_O was prepared (Scheme 1).

This approach was extended to the functionalisation of a second base-resin, mesoporous polydivinylbenzene (pDVB), which was prepared according to literature methods [17]. Although it has a chemical structure closely related to that of conventional pS-DVB resins in the dry-state, it features both a very large pore volume and a very high specific surface area (up to 1000 m^2^/g), for about its half arising from mesopores in the diameter range 2–50 nm [17]. These morphological features [17,24,25], already well established in the literature, are confirmed herein by the N_2_ physisorption data for a 1% palladium catalyst supported on a sulfonated form of pDVB (Table 1).

In fact, this material is essentially mesoporous, with small contributions from micro- (d < 2 nm) and macropores (d > 50 nm). The acylated material, pDVB_C_4_F_7_O, has a slightly smaller pore volume, which is compatible with some partial hindering of the pores because of the presence of the fluorous ponytails. It is still an essentially mesoporous material in that its mesopores represent more than 50% of the total pore volume. The presence of a relatively large fraction of macropores suggests that the polymer framework was damaged by the acylation, carried out under strongly acidic conditions for 48 h.

The acylation degrees determined on the basis of both the elemental analysis and the mass balance of the resins recovered after the acylation are reported in Table 2.

The mass balance provides an acylation degree slightly lower than the elemental analysis, most probably due to the difficulty of quantitative recovery of the resins from their multi-step preparation, which leads to the underestimation of the increase in their weights.

**Figure 1:**
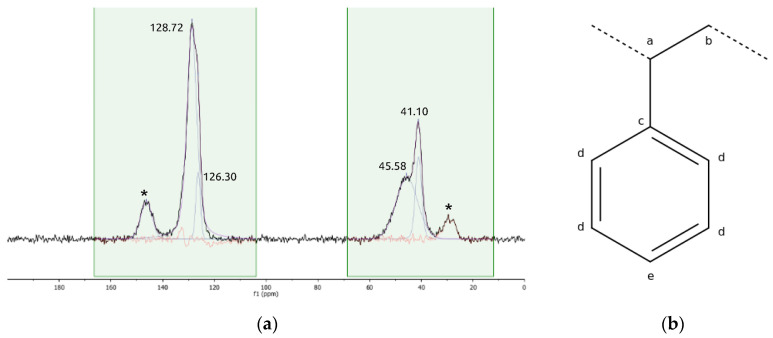
{^1^H}-^13^C CP-MAS spectrum (**a**) of the pristine gel-type resin (contact time 1 ms, MAS rate = 10 kHz, T = 25 °C); asterisks indicate the spinning sidebands and graphic representation (**b**) of the inequivalent carbon atoms of the repeating unit (for the sake of simplicity the cross-linker unit, ca. 2% mol/mol, is omitted).

**Figure 2:**
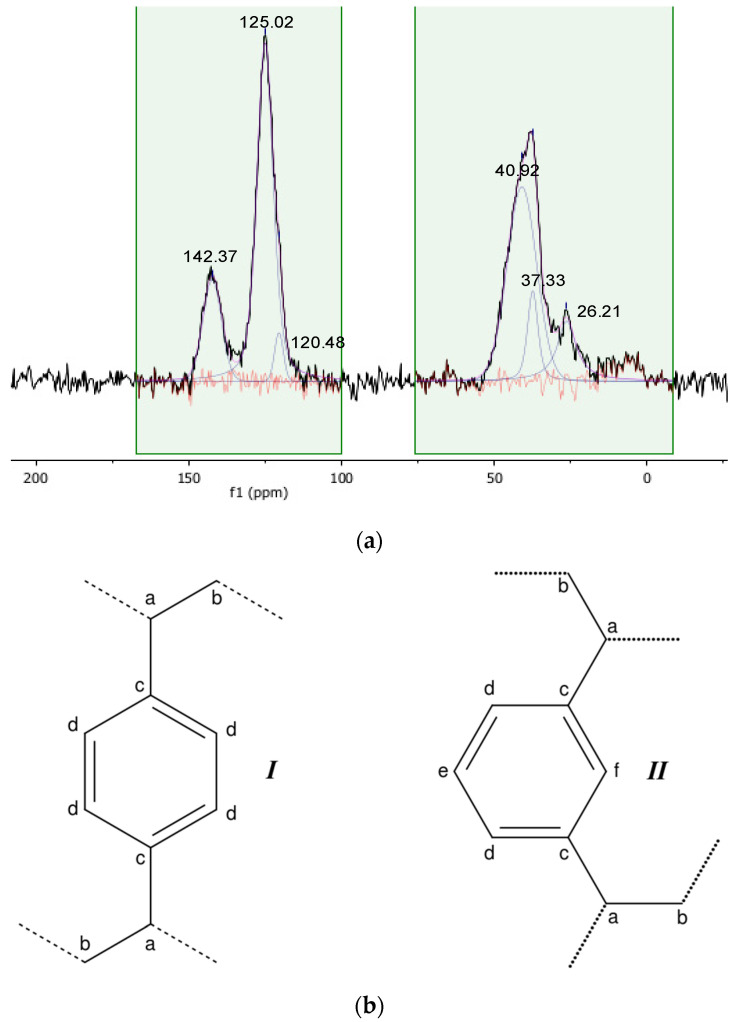
{^1^H}-^13^C CP-MAS spectrum (**a**) of the pristine pDVB resin (contact time 1 ms, MAS rate = 10 kHz, T = 25 °C) and graphic representation (**b**) of the inequivalent carbon atoms of the two most abundant repeating units.

For each material, the starting cross-linked polymer has been characterised by solid-state NMR spectroscopy (Figure 1a and Figure 2a). The resonances of each magnetically inequivalent carbon atom have been analysed by signal deconvolution, and the spinning sidebands have been assigned by comparison of spectra registered at different spinning rates.

The signals at 41.1 ppm and 45.6 ppm in the spectrum of the gel-type parent resin (Figure 1a) can be assigned to the backbone carbon atoms (a, b; Figure 1b). The signal at 146.3 ppm is due to the aromatic carbon atoms directly bound to the polymer chains (c; Figure 1b), and the signals at 128.7 and 126.3 ppm can be attributed to the non-functionalised aromatic carbon atoms (d, e; Figure 1b) [26].

In the spectrum of the parent pDVB (Figure 2a), the signals of the backbone carbons of the aromatic carbon atoms directly bound to the polymeric chains (c; Figure 2b) and of the non-functionalised aromatic carbon atoms (d, e; Figure 1b) are observed at 40.9 and 37.3 ppm (a, b), at 142.4 ppm (c), and at 125.0 and 120.5 ppm (d, e, f) [27,28]. No signals from vinylic C atoms belonging to free double C-C bonds were detected, although they were reported for a different kind of pDVB at 111.4 and 137.5 ppm [24]. This shows that the synthesis of mesoporous pDVB [17] takes up all the vinyl groups of the monomer. Although this process is nominally a homo-polymerization of DVB, the commercially available technical grade monomer was used. It is a mixture of meta- and para-DVB, which also contains ca. 20% of ethylstyrene. Thus, the number of possible repeating units of pDVB from technical DVB is actually four: two are the cross-links represented in Figure 2b (I and II). The other two has an ethyl group per aromatic ring as a substituent instead of one of the polymeric chains and for the present discussion it is assumed that an ethyl group and a polymeric chain have similar effects as substituents of the aromatic rings of pDVB. As these ethyl-substituted monomeric units are non-crosslinking, the actual cross-linking degree is lower than 100%. The meta/para ratio (m/p) in technical grade DVB is generally close to 2/1 [29], and this was confirmed by the ^1^H-NMR spectrum of DVB used in this work. It showed the presence of ca. 20% of ethylstyrene and a 65/35 proportion of meta- and para-DVB. Even taking into only the repeating units I and II depicted in Figure 2b, at least four kinds of inequivalent aromatic carbon atoms can be identified (c, d, e, f; Figure 2b), with respective relative abundances of 2, 2.7, 0.65, and 0.65. At best, only three peaks can be extracted by the deconvolution of the overlapped signals, which indicates that it is not possible to fully discriminate them under these experimental conditions.

**Figure 3:**
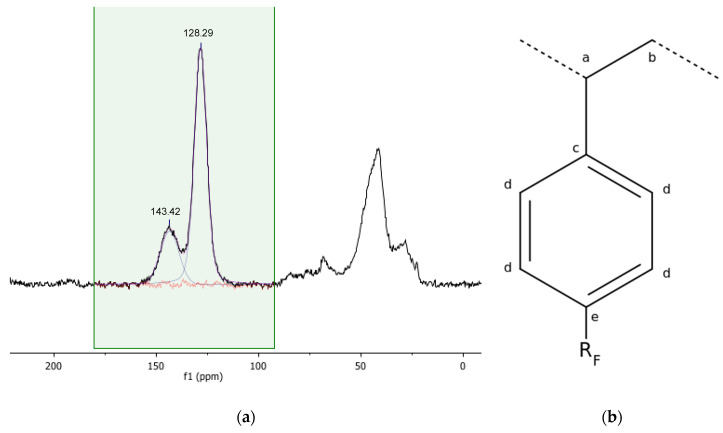
{^1^H}-^13^C CP-MAS spectrum (**a**) of Gel_C_4_F_7_O (contact time 1 ms, MAS rate = 10 kHz, T = 25 °C) and graphic representation (**b**) of the inequivalent carbon atoms of the monomeric unit bearing the perfluorobutirroyl group.

**Figure 4:**
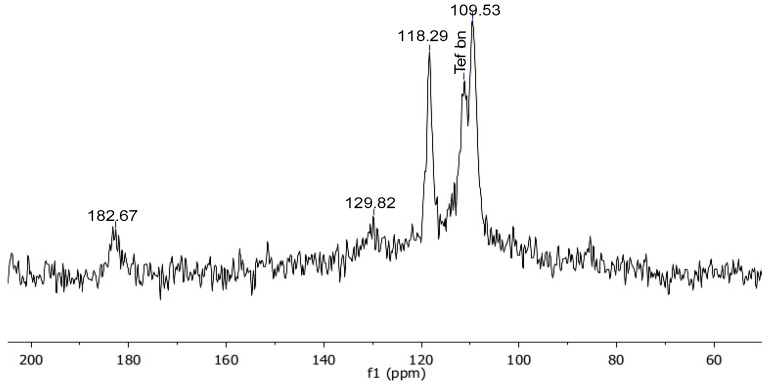
{^19^F}-^13^C CP-MAS spectrum of Gel_C_4_F_7_O (contact time 2 ms, MAS rate = 10 kHz, T = 25 °C; the signal labelled as “Teflon” arises from the rotor plugs).

**Figure 5:**
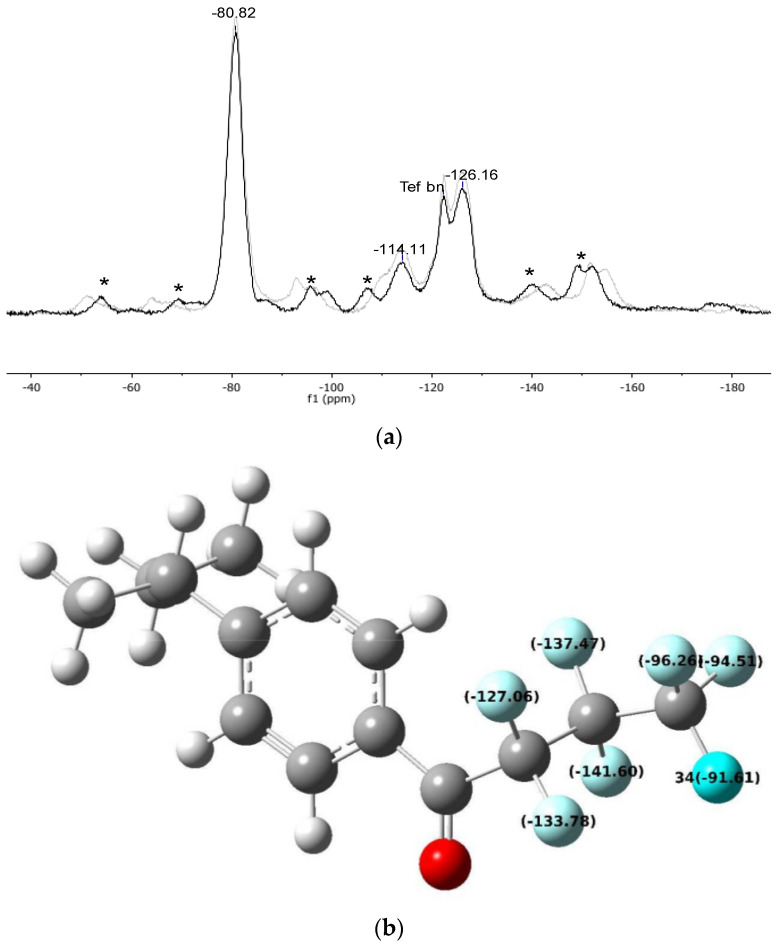
^19^F-MAS spectrum (**a**) of Gel_C_4_F_7_O at MAS rate = 10 kHz (black line) and MAS rate = 11 kHz (grey line; the signal labelled as “Teflon” arises from the rotor plugs; asterisks indicate the spinning sidebands) and DFT calculations (**b**) for FBMPB.

**Figure 6:**
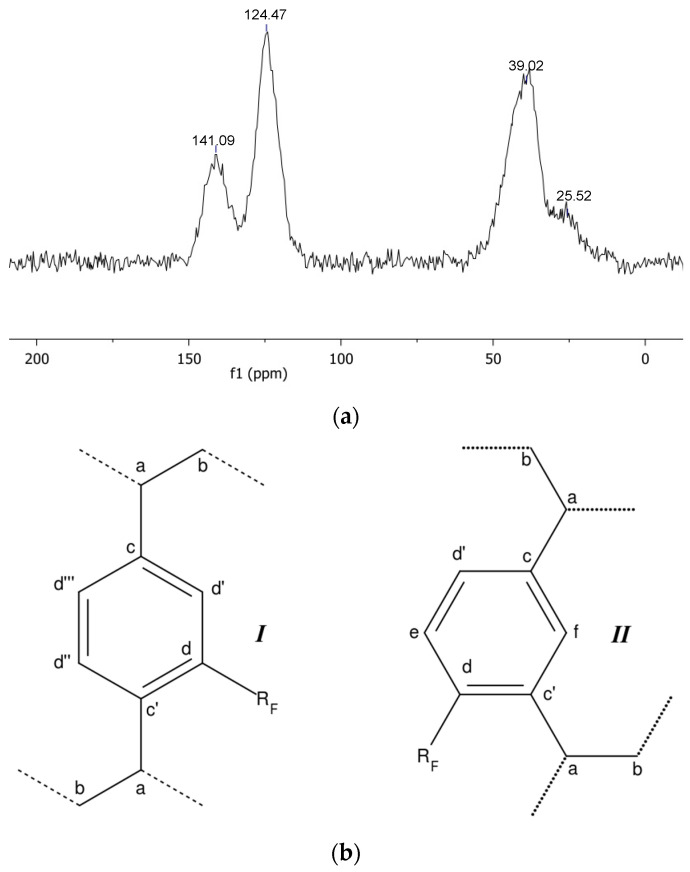
{^1^H}-^13^C-CP MAS NMR spectrum (**a**) of the acylated pDVB (*pDVB_C_4_F_7_O*; contact time 1 ms, MAS rate = 10 kHz, T = 25 °C) and graphic representation (**b**) of the inequivalent carbon atoms of two most abundant repeating units (***I***, ***II***).

**Figure 7:**
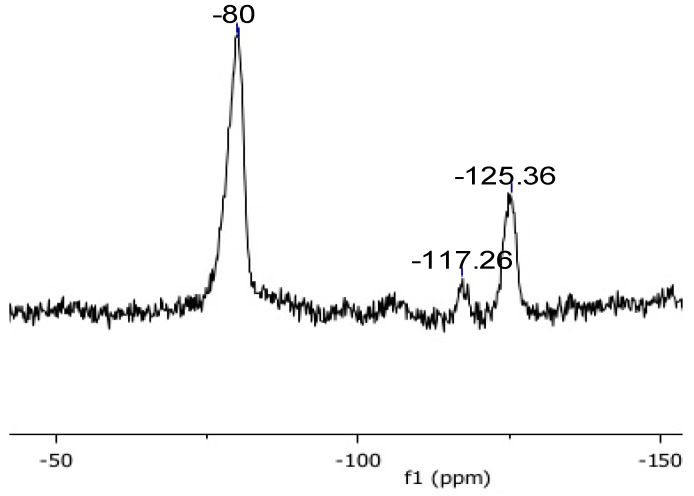
^19^F-MAS spectrum of pDVB (pDVB_C_4_F_7_O). MAS rate = 10 kHz.

The NMR characterisation of Gel_C_4_F_7_O and of pDVB_C_4_F_7_O included both their solid-state ^13^C and the ^19^F spectra (Figure 3, Figure 4, Figure 5, Figure 6 and Figure 7). The ^13^C spectra of the former were cross-polarized from either ^1^H or ^19^F (Figure 3 and Figure 4); the ^19^F-MAS NMR spectrum is illustrated in Figure 5. Only the {^1^H}-^13^C-CP MAS NMR spectrum was collected for *pDVB_C_4_F_7_O* (Figure 6), and its ^19^F-MAS NMR spectrum is illustrated in Figure 7.

The attributions of the signals observed in the {^1^H}-^13^C-CP MAS NMR spectra of the acylated resins are summarised in Table 3.

In the case of Gel_C_4_F_7_O, only two kinds of inequivalent aromatic C atoms are observed (Figure 3 and Table 3) in the {^1^H}-^13^C-CP MAS NMR spectrum, in spite of three expected signals (c, d, e; Figure 3b). However, as discussed above, not all the overlapped signals could be resolved by deconvolution. In this case, it must also be taken into account that the abundance of the acyl-substituted C atoms (e) is low (the acylation degree is well below 100%) and that the linewidth in the {^1^H}-^13^C-CP MAS NMR spectra of both the acylated derivatives is higher than in the spectra of the parent resins. Indeed, the latter finding indicates that the polymer framework is more disordered after the acylation, which is another indirect hint of functionalisation. Two signals from aliphatic carbon atoms (43.03 ppm, a; 28.33 ppm, b) are observed, which can be readily attributed to the methine and methylene C atoms of the polymeric chains (a, b; Figure 3b).

The general electronic and steric features of the electrophilic aromatic substitutions militate in favour of a para-acylation, and this is confirmed by the {^9^F}-^13^C-CP MAS spectrum of the acylated gel-type resin (Figure 4; the signal attributions are collected in Table 4).

In this spectrum, the carbon atoms of the perfluorobutirroyl groups can be detected, including the carbonylic carbon atom at 182.67 ppm.

The signal at 129.82 ppm is particularly interesting. Since it is too weak to be attributed to the acylic chain, it must arise from an aromatic carbon atom, most likely the one substituted by the perfluorobutyryl groups (the cross-polarization builds up from neighbouring atoms). The para-aromatic carbon atom (e) resonates almost at the same chemical shift (128.29 ppm) in the {^1^H}-^13^C-CP-MAS of this resin, and this finding supports the hypothesis that the acylation of the aromatic rings of the gel resins took place in para position with respect to the polymeric chains. It also shows that the observed fluorine signals did not arise from perfluorinated butyric acid physically embedded in the resin.

The signals of the ^19^F-MAS spectrum of *Gel_CF_4_H_7_O* can be attributed upon comparison with the literature spectrum of perfluorobutyryl chloride [30]. Further support to the attribution of the ^19^F-MAS NMR signals was obtained from DFT calculations on the molecule of 4-heptafluorobutyryl-(1-methylpropyl)benzene (FBMPB) (Figure 5b and Table 5, see Experimental section for details). For a reliable comparison, the differences between the DFT values and the experimental ones (reported as “Δ” in Table 5) should be considered. It is anticipated here that these differences are very similar for each type of magnetically equivalent fluorine atoms in both the acylated resins, which indicates a systematic offset between the experimental and calculated data.

The ^13^C-MAS NMR spectrum of *pDVB_C_4_F_7_O*, obtained by cross-polarization from the protons, is illustrated in Figure 6, and the attributions of its signals are summarised in Table 3.

Additionally, for this material, only two peaks from the aromatic C atoms could be detected, although none of them is equivalent to each other (see Figure 6b). Therefore, this spectrum does not provide conclusive evidence on the position of the acylic groups. However, in the repeating unit ***I***, there is only one possible position for the acyl group because, in pDVB, all its unsubstituted aromatic C atoms are equivalent. In the repeating unit ***II*** of pDVB (the most abundant one, see above), the attack of the acyl group to the equivalent positions d and d’ (Figure 2b) should be favoured by the electron-donating effect of the alkyl substituents (the polymeric chains) to the ring, as both are ortho- or para-positions to the substituted carbon atoms. The acylation in the position f should also be favoured by the electronic effects of the substituents (it is ortho to both), but it is expectedly hindered by strong steric repulsion. the position e is the most favoured from the point of view of steric effects, but it is meta to both the substituents hence the least favoured by the electronic effects. Taking into account that the acylation degree is again well below 100% (actually about half as much as in Gel_C_4_F_7_O), the number of possible different sites for acylation in pDVB and a balance between electronic and steric factors, the most likely positions of the perfluorobutyryl groups in pDVB_C_4_F_7_O should be those indicated in Figure 6b. In summary, we tentatively propose that the acyl groups in the aromatic rings of the cross-linking units which are in ortho position with respect to one of poymeric chains and in para- to the other one.

The ^19^F-MAS NMR spectrum of pDVB_C_4_F_7_O (Figure 7 and Table 5) is similar to the one of Gel_C_4_F_7_O; and the resonances of the fluorine atoms are observed at almost the same chemical shifts. However, the signal-to-noise ratio is much lower in the case of pDVB_C_4_F_7_O, less dense than Gel_C_4_F_7_O (it is much more porous) and with a lower fluorination degree. In consequence, the amount of resonating nuclei is lower, and this also prevented the collection of the {^19^F}-^13^C-CP-MAS NMR spectrum for this material.

To evaluate the effect of the functionalisation of the polymer matrix with the fluoroacylic groups, we prepared ion-exchange acid catalysts (IECs) by sulfonation of pDVB_C_4_F_7_O and Gel_C_4_F_7_O with concentrated sulfuric acid and tested them in the esterification of stearic acid (SA) with methanol. This reaction represents the first step of the production of biodiesel from waste oils, usually containing a remarkable amount (up to 15–20%) [14,22] of free fatty acids. In general, the performance of IECs is strongly affected by the swelling behaviour of the polymer matrix because, in this reaction, the substrates, the fatty acid, and methanol compete for the occupation of the catalytic sites. Moreover, the polar sulfonic groups of these catalysts can be effectively solvated by water, which is a co-product of the reaction. This interaction favours its close to the catalytic sites and can contribute to keeping the mostly hydrophobic molecules of the fatty acid away from them. In this context, the presence of the hydrophobic fluorinated acyl groups is expected to have a beneficial effect on the catalytic performance, either by facilitating the ingress of SA in the catalyst through “assisted absorption” or by expelling the produced water [12,13].

**Figure 8:**
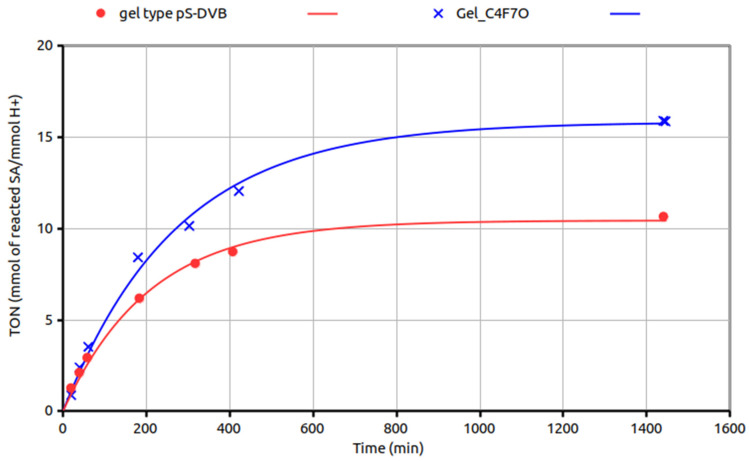
Turnover number (TON, mmol of consumed stearic acid/mmol of H^+^) as a function of time of Gel_C_4_F_7_O (blue crosses) and of its parent gel-type resin (red circles), both sulfonated with sulfuric acid (ion exchange capacity = 2.33 and 4.93 mmol H^+^/g, respectively) in the esterification of SA (8 g) with methanol (20 g) in vegetable oil (72 g); 0.5 g of catalyst; T = 65 °C).

The results of a comparative test of esterification with methanol of a stearic acid solution in vegetable oil with acid catalysts based on Gel_C_4_F_7_O and on its parent gel type are summarised in Figure 8.

The final turnover number increases by 50%, and it also changes faster with time upon acylation. This signifies that SA is consumed with a turnover frequency generally higher for Gel_C_4_F_7_O from the beginning to the end of the experiment. This suggests that the fluoroacylation helps in keeping a relatively high concentration of SA in the catalyst and/or in limiting the build-up of water in the catalyst, especially at a relatively high conversion, when their amounts in the reaction mixture are, respectively, vanishing and increasing. A high concentration of water in the catalyst would be detrimental because it would shift the equilibrium back to the alcohol-acid mixture and could hydrolyse the sunflower oil used as the solvent as well. Both the swollen gel-type catalysts, however, are soft materials with relatively little mechanical stability. Thus, under the magnetic stirring conditions employed herein, their extensive grinding makes their recovery and recycling a difficult operation.

## 4. Conclusions

Styrenic cross-linked polymers can be readily acylated with perfluorinated acyls to a considerable degree, as confirmed by {^1^H}-^13^C CP-MAS NMR, {^19^F}-^13^C CP-MAS NMR, and ^19^F-MAS NMR. The resulting materials can be sulfonated to form acidic ion-exchange catalysts with enhanced hydrophobicity, as suggested by their improved performance in the esterification of stearic acid with methanol in comparison with conventional sulfonated polystyrene-divinylbenzene resins. Further work is now needed to check for the scope of this approach and to ascertain whether the prevailing effect in the activation of the sulfonated ion-exchange catalysts upon acylation with fluorinated acyls is the “assisted absorption” of SA and/or the enhanced expulsion of water.

## Figures and Tables

**Table 1:** Pore volume (cm^3^·g^−1^) and its distribution (%) in catalysts obtained from pDVB.

	Macro	Meso	Micro	V_p_
Pd-SpDVB	10%	81%	9%	0.85
pDVB_C_4_F_7_O	39%	54%	7%	0.72

**Table 2:** Elemental analysis and acylation degree of acylated resins.

	C (%, *w*/*w*)	H (%, *w*/*w*)	Fraction of Acylated Rings (%)	
			b	a
Gel_C_4_F_7_O	76.36	6.05	19	21
pDVB_C_4_F_7_O	78.69	7.05	10	13

a: from elemental analysis; b: from mass balance.

**Table 3:** Attribution of the signals in the {^1^H}-^13^C-CP MAS spectrum of *pDVB_C_4_F_7_O* (Figure 5 and Figure 6) of acylated resins (Figure 7).

	Ca	Cb	Cc	Cd,e
Gel_C_4_F_7_O	43.03 ppm	28.33 ppm	143.42 ppm	128.29 ppm
pDVB_C_4_F_7_O	39.02 ppm	25.52 ppm	141.09 ppm	124.47 ppm

**Table 4:** Attribution of the signals of the {^19^F}-^13^C-CP-MAS spectrum of Gel_C_4_F_7_O.

Resin-**C**O-CF2-CF2-CF3	182.67 ppm
Resin-CO-**C**F2-**C**F2-CF3	129.82 ppm
Resin-CO-CF2-CF2-**C**F3	118.47 ppm

**Table 5:** Attribution of the signals of the ^19^F-MAS NMR spectra of Gel_C_4_F_7_O and pDVB_C_4_F_7_O. The DFT data are average values calculated for each fluorine atom.

	Resin-CO-CF_2_-CF_2_-CF_3_	Resin-CO-CF_2_-CF_2_-CF_3_	Resin-CO-CF_2_-CF_2_-CF_3_
FBMPB ^a^	−130.42	−139.54	−93.94
Gel_C_4_F_7_O	−114.11	−126.16	−80.82
pDVB_C_4_F_7_O	−117.26	−125.36	−80
Δ (Gel_C_4_F_7_O)	16.31	13.38	13.12
Δ (pDVB_C_4_F_7_O)	13.16	14.18	13.94

a: from DFT calculation.

## Data Availability

No new data were created.

## References

[1] Dorfner K. (2011). Ion Exchangers.

[2] Sherrington D.C., Hodge P. (1988). Syntheses and Separations Using Functional Polymers.

[3] Maul J., Frushour B.G., Kontoff J.R., Eichenauer H., Ott K.-H., Schade C. (2007). Polystyrene and Styrene Copolymers. Ullmann’s Encyclopedia of Industrial Chemistry.

[4] de Dardel F., Arden T.V. (2008). Ion Exchangers. Ullmann’s Encyclopedia of Industrial Chemistry.

[5] Corain B., Zecca M., Canton P., Centomo P. (2010). Synthesis and Catalytic Activity of Metal Nanoclusters inside Functional Resins: An Endeavour Lasting 15 Years. Philos. Trans. R. Soc. Math. Phys. Eng. Sci..

[6] Marco Z., Paolo C., Benedetto C., Corain B., Schmid G., Toshima N. (2008). CHAPTER 10—Metal Nanoclusters Supported on Cross-Linked Functional Polymers: A Class of Emerging Metal Catalysts. Metal Nanoclusters in Catalysis and Materials Science.

[7] De Zan L., Gasparovicova D., Kralik M., Centomo P., Carraro M., Campestrini S., Jerabek K., Corain B. (2007). Nanoclustered Palladium(0) Supported on a Gel-Type Poly-Acrylonitrile–N,N-Dimethylacrylamide–Ethylenedimethacrylate Resin: Nanostructural Aspects and Catalytic Behaviour. J. Mol. Catal. Chem..

[8] Centomo P., Zecca M., Corain B. (2007). Template Controlled Synthesis (TCS) of Size-Controlled Metal Nanoclusters: Preparation of Nanostructured Metals Supported by Inorganic Supports. J. Clust. Sci..

[9] Centomo P., Jeřábek K., Canova D., Zoleo A., Maniero A.L., Sassi A., Canton P., Corain B., Zecca M. (2012). Highly Hydrophilic Copolymers of N,N-Dimethylacrylamide, Acrylamido-2-Methylpropanesulfonic Acid, and Ethylenedimethacrylate: Nanoscale Morphology in the Swollen State and Use as Exotemplates for Synthesis of Nanostructured Ferric Oxide. Chem.—Eur. J..

[10] Mbaraka I.K., Shanks B.H. (2005). Design of Multifunctionalized Mesoporous Silicas for Esterification of Fatty Acid. J. Catal..

[11] Harmer M.A., Sun Q. (2001). Solid Acid Catalysis Using Ion-Exchange Resins. Appl. Catal. Gen..

[12] Jerabek K., Hankova L., Holub L., Corain B., Zecca M., Centomo P., Bonato I. (2012). Strongly Acidic Ion Exchanger Catalyst and Method of Preparing the Same.

[13] Centomo P., Bonato I., Hanková L., Holub L., Jeřábek K., Zecca M. (2013). Novel Ion-Exchange Catalysts for Reactions Involving Lipophilic Reagents: Perspectives in the Reaction of Esterifications of Fatty Acids with Methanol. Top. Catal..

[14] Gallezot P. (2012). Conversion of Biomass to Selected Chemical Products. Chem. Soc. Rev..

[15] Soltani S., Rashid U., Al-Resayes S.I., Nehdi I.A. (2017). Recent Progress in Synthesis and Surface Functionalization of Mesoporous Acidic Heterogeneous Catalysts for Esterification of Free Fatty Acid Feedstocks: A Review. Energy Convers. Manag..

[16] Riess J.G., Blanc M.L. (1982). Solubility and Transport Phenomena in Perfluorochemicals Relevant to Blood Substitution and Other Biomedical Applications. Pure Appl. Chem..

[17] Zhang Y., Wei S., Liu F., Du Y., Liu S., Ji Y., Yokoi T., Tatsumi T., Xiao F.-S. (2009). Superhydrophobic Nanoporous Polymers as Efficient Adsorbents for Organic Compounds. Nano Today.

[18] Frisch M.J., Trucks G.W., Schlegel H.B., Scuseria G.E., Robb M.A., Cheeseman J.R., Scalmani G., Barone V., Petersson G.A., Nakatsuji H. (2016). Gaussian 16 Rev. B.01.

[19] Bagno A., Rastrelli F., Saielli G. (2003). Predicting 13C NMR Spectra by DFT Calculations. J. Phys. Chem. A.

[20] Bagno A., Rastrelli F., Saielli G. (2006). Toward the Complete Prediction of the 1H and 13C NMR Spectra of Complex Organic Molecules by DFT Methods: Application to Natural Substances. Chem.—Eur. J..

[21] Jerabek K. (1985). Characterization of Swollen Polymer Gels Using Size Exclusion Chromatography. Anal. Chem..

[22] Jeřábek K., Hanková L., Holub L. (2010). Working-State Morphologies of Ion Exchange Catalysts and Their Influence on Reaction Kinetics. J. Mol. Catal. Chem..

[23] Jeřábek K., Zecca M., Centomo P., Marchionda F., Peruzzo L., Canton P., Negro E., Di Noto V., Corain B. (2013). Synthesis of Nanocomposites from Pd0 and a Hyper-Cross-Linked Functional Resin Obtained from a Conventional Gel-Type Precursor. Chem.—Eur. J..

[24] Liu F., Meng X., Zhang Y., Ren L., Nawaz F., Xiao F.-S. (2010). Efficient and Stable Solid Acid Catalysts Synthesized from Sulfonation of Swelling Mesoporous Polydivinylbenzenes. J. Catal..

[25] Hanková L., Holub L., Jeřábek K. (2015). Formation of Porous Polymer Morphology by Microsyneresis during Divinylbenzene Polymerization. J. Polym. Sci. Part B Polym. Phys..

[26] Law R.V., Sherrington D.C., Snape C.E., Ando I., Korosu H. (1995). Solid State 13C MAS NMR Studies of Anion Exchange Resins and Their Precursors. Ind. Eng. Chem. Res..

[27] Ford W.T., Periyasamy M., Mohanraj S., McEnroe F.J. (1989). Peak Area Measurements of Cross-Polarization Magic-Angle-Spinning 13C-NMR Spectra of Crosslinked Polystyrenes. J. Polym. Sci. Part Polym. Chem..

[28] Law R.V., Sherrington D.C., Snape C.E. (1997). Quantitative Solid State 13C NMR Studies of Highly Cross-Linked Poly(Divinylbenzene) Resins. Macromolecules.

[29] Tanaka S., Matsumoto M., Goseki R., Ishizone T., Hirao A. (2013). Living Anionic Polymerization of 1,4-Divinylbenzene and Its Isomers. Macromolecules.

[30] Zhao C., Zhou R., Pan H., Jin X., Qu Y., Wu C., Jiang X. (1982). Thermal Decomposition of Some Perfluoro- and Polyfluorodiacyl Peroxides. J. Org. Chem..

